# Supplementary material for: Systematic Review and Meta-Analysis of Risk Factors for Dehydration and the Development of a Predictive Scoring System
Source: Healthcare (Basel). 2025 Aug 12;13(16):1974. doi: 10.3390/healthcare13161974 (PMC12385403; doi:10.3390/healthcare13161974)
Supplement: Supplementary file 1 [file healthcare-13-01974-s001.zip › healthcare-3746273-supplementary.pdf]

Manuscript Checklist

Section and Topic	Item #	Checklist item	Location where item is reported
**TITLE**			
Title	1	Identify the report as a systematic review.	Title Page (Page 1)
**ABSTRACT**			
Abstract	2	Provide a structured summary following PRISMA Abstract guidelines.	Abstract (Page 1)
**INTRODUCTION**			
Rationale	3	Describe the rationale for the review in the context of existing knowledge.	Section 1.2 Rationale (Pages 2)
Objectives	4	Provide an explicit statement of the objective(s) or question(s) the review addresses.	Section 1.3 Objectives of the Research (Page 2)
**METHODS**			
Eligibility criteria	5	Specify the inclusion and exclusion criteria for the review and how studies were grouped.	Section 2.2 Criteria for Eligibility (Pages 3)
Information sources	6	Specify all databases, registers, websites, organisations, reference lists and other sources searched or consulted to identify studies. Specify the date when each source was last searched or consulted.	Section 2.3 Information Sources (Page 3)
Search strategy	7	Present the full search strategies for all databases, registers and websites, including any filters and limits used.	Section 2.4 Search Strategy (Page 4)
Selection process	8	Specify the methods used to decide whether a study met the inclusion criteria of the review, including how many reviewers screened each record and each report retrieved, whether they worked independently, and if applicable, details of automation tools used in the process.	Section 2.5 Study Selection (Pages 5), PRISMA Flow Diagram (Page 5)
Data collection process	9	Specify the methods used to collect data from reports, including how many reviewers collected data from each report, whether they worked independently, any processes for obtaining or confirming data from study investigators, and if applicable, details of automation tools used in the process.	Section 2.6 Data Extraction (Pages 6)
Data items	10a	List and define all outcomes for which data were sought. Specify whether all results that were compatible with each outcome domain in each study were sought (e.g. for all measures, time points, analyses), and if not, the methods used to decide which results to collect.	Section 2.6.3 Outcome Measures (Pages 6)
	10b	List and define all other variables for which data were sought (e.g. participant and intervention characteristics, funding sources). Describe any assumptions made about any missing or unclear information.	Section 2.6.2 Risk Factors Assessed (Pages 6), Section 2.6.4 Data Extraction Forms (Page 6)
Study risk of bias assessment	11	Specify the methods used to assess risk of bias in the included studies, including details of the tool(s) used, how many reviewers assessed each study and whether they worked independently, and if applicable, details of automation tools used in the process.	Section 2.7 Quality Assessment (Pages 7), Table 2 (Page 9)
Effect measures	12	Specify for each outcome the effect measure(s) (e.g. risk ratio, mean difference) used in the synthesis or presentation of results.	Section 2.8 Data Synthesis and Meta-Analysis (Pages 10)
Synthesis methods	13a	Describe the processes used to decide which studies were eligible for each synthesis (e.g. tabulating the study intervention characteristics and comparing against the planned groups for each synthesis (item #5)).	Section 2.5 Study Selection (Pages 4), Section 2.8 Data Synthesis and Meta-Analysis (Page 10), PRISMA Flow Diagram (Page 5)
	13b	Describe any methods required to prepare the data for presentation or synthesis, such as handling of missing summary statistics, or data conversions.	Section 2.8 Data Synthesis and Meta-Analysis (Pages 10)

Manuscript Checklist

Section and Topic	Item #	Checklist item	Location where item is reported
	13c	Describe any methods used to tabulate or visually display results of individual studies and syntheses.	Section 3.4 Meta-Analysis (Pages 12), Table 4 (Pages 13)
	13d	Describe any methods used to synthesize results and provide a rationale for the choice(s). If meta-analysis was performed, describe the model(s), method(s) to identify the presence and extent of statistical heterogeneity, and software package(s) used.	Section 2.8 Data Synthesis and Meta-Analysis (Pages 10)
	13e	Describe any methods used to explore possible causes of heterogeneity among study results (e.g. subgroup analysis, meta-regression).	Section 2.8.2 Handling Heterogeneity (Pages 10), Section 3.4 Meta-Analysis (Pages 8-9), Table 3 (Page 11)
	13f	Describe any sensitivity analyses conducted to assess robustness of the synthesized results.	Section 3.4.2 Sensitivity Analysis (Pages 9-10)
Reporting bias assessment	14	Describe any methods used to assess risk of bias due to missing results in a synthesis (arising from reporting biases).	Section 2.8.3 Evaluation of Publication Bias (Pages 8-9)
Certainty assessment	15	Describe any methods used to assess certainty (or confidence) in the body of evidence for an outcome.	Section 2.7 Quality Assessment (Pages 7-8), Section 3.4 Meta-Analysis (Pages 12), Sensitivity Analysis (Pages 12)
**RESULTS**			
Study selection	16a	Describe the results of the search and selection process, from the number of records identified in the search to the number of studies included in the review, ideally using a flow diagram.	Section 3.1 Study Selection (Pages 5-6), PRISMA Flow Diagram (Page 6)
	16b	Cite studies that might appear to meet the inclusion criteria, but which were excluded, and explain why they were excluded.	Section 3.1 Study Selection (Pages 11)
Study characteristics	17	Cite each included study and present its characteristics.	Section 3.2 Study Characteristics (Pages 11), Table 3 (Page 11)
Risk of bias in studies	18	Present assessments of risk of bias for each included study.	Section 3.3 Quality Assessment (Pages 12), Table 2 (Page 9)
Results of individual studies	19	For all outcomes, present, for each study: (a) summary statistics for each group (where appropriate) and (b) an effect estimates and its precision (e.g. confidence/credible interval), ideally using structured tables or plots.	Section 3.4 Meta-Analysis (Pages 8-9), Table 3 (Page 10)
Results of syntheses	20a	For each synthesis, briefly summarise the characteristics and risk of bias among contributing studies.	Section 3.4 Meta-Analysis (Pages 12), Table 3 (Page 11)
	20b	Present results of all statistical syntheses conducted. If meta-analysis was done, present for each the summary estimate and its precision (e.g. confidence/credible interval) and measures of statistical heterogeneity. If comparing groups, describe the direction of the effect.	Section 3.4 Meta-Analysis (Pages 12)
	20c	Present results of all investigations of possible causes of heterogeneity among study results.	Section 3.4 Meta-Analysis (12), and 3.4.2 Subgroup Analysis (Page 12)
	20d	Present results of all sensitivity analyses conducted to assess the robustness of the synthesized results.	Section 3.4 Sensitivity Analysis (12)
Reporting biases	21	Present assessments of risk of bias due to missing results (arising from reporting biases) for each synthesis assessed.	Section 2.8.3 Evaluation of Publication Bias (10)
Certainty of	22	Present assessments of certainty (or confidence) in the body of evidence for each outcome	Section 3.4 Sensitivity Analysis (12)

Manuscript Checklist

Section and Topic	Item #	Checklist item	Location where item is reported
evidence		assessed.	
**DISCUSSION**			
Discussion	23a	Provide a general interpretation of the results in the context of other evidence.	Section 4.1 Summary of Findings (Page 16)
	23b	Discuss any limitations of the evidence included in the review.	Section 4.4 Study Strengths and Limitations (Page 17)
	23c	Discuss any limitations of the review processes used.	Section 4.4 Study Strengths and Limitations (Page 11)
	23d	Discuss implications of the results for practice, policy, and future research.	Section 4.3 Implications for Practice (Page 10), Section 4.5 Future Research (Page 16)
**OTHER INFORMATION**			
Registration and protocol	24a	Provide registration information for the review, including register name and registration number, or state that the review was not registered.	Section 2.1 Protocol and Registration (Page 2)
	24b	Indicate where the review protocol can be accessed, or state that a protocol was not prepared.	Section 2.1 Protocol and Registration (Page 2)
	24c	Describe and explain any amendments to information provided at registration or in the protocol.	No amendments were made to the registered protocol after the initial submission."
Support	25	Describe sources of financial or non-financial support for the review, and the role of the funders or sponsors in the review.	This research did not receive any specific grant from funding agencies in the public, commercial, or not-for-profit sectors. The authors conducted this study independently without external financial support.
Competing interests	26	Declare any competing interests of review authors.	The authors declare no conflicts of interest in relation to this study.
Availability of data, code and other materials	27	Report which of the following are publicly available and where they can be found template data collection forms; data extracted from included studies; data used for all analyses; analytic code; any other materials used in the review.	The datasets generated and analyzed during this study are not publicly available but are available from the corresponding author upon reasonable request.
